# The Elusive Role of the Prion Protein and the Mechanism of Toxicity in Prion Disease

**DOI:** 10.1371/journal.ppat.1004745

**Published:** 2015-05-07

**Authors:** Roberto Chiesa

**Affiliations:** Department of Neuroscience, IRCCS–Istituto di Ricerche Farmacologiche Mario Negri, Milan, Italy; Washington University School of Medicine, UNITED STATES

PrP^Sc^, a misfolded, aggregation-prone isoform of the cellular prion protein (PrP^C^), is the infectious prion agent responsible for incurable brain diseases such as scrapie of sheep, bovine spongiform encephalopathy, and its human counterpart, variant Creutzfeldt-Jakob disease. In these disorders, collectively known as prion diseases, exogenous PrP^Sc^ propagates in the infected host by imprinting its aberrant conformation onto endogenous PrP^C^, eventually triggering a rapidly progressing neurodegenerative process that invariably leads to death. But what is the function of PrP^C^ besides serving as a substrate for the generation of PrP^Sc^? And how does PrP^C^ misfolding cause neurological disease?

## The Cellular Prion Protein

PrP^C^ is a cell surface glycoprotein expressed in neurons and many other body cells. It is synthesized in the endoplasmic reticulum (ER), where it undergoes oxidative folding, N-linked glycosylation, and addition of a glycosyl-phosphatidyl-inositol (GPI) anchor that attaches the protein’s C terminus to the lipid bilayer. After transit in the Golgi, PrP^C^ is delivered to the plasma membrane, where it resides in lipid rafts, which are membrane microdomains rich in cholesterol and sphingolipids. Some PrP^C^ molecules are constitutively endocytosed and either recycled to the plasma membrane or delivered to lysosomes for degradation. PrP^C^ has a flexible N terminus (residues 23–127, mouse PrP numbering) that can interact with copper and zinc ions, and a C-terminal globular domain (residues 128–231) comprising three α-helices and two short anti-parallel β-strands.

Inactivation of the PrP^C^ gene in living organisms produced variable phenotypes. Knockdown of the PrP^C^-related genes *PrP-1* and *PrP-2* in the zebrafish *Danio rerio* caused, respectively, gastrulation arrest and malformed brains and eyes, indicative of essential roles in the fish’s development [1,2]. In contrast, PrP^C^ knockout mice and cows had no major developmental or neuroanatomical defects (reviewed in [3]), indicating non-essential or redundant functions in higher vertebrates.

Based on the analysis of mild phenotypic traits that develop in PrP^C^ knockout mice and on cell culture studies, mammalian PrP^C^ has been assigned roles in many biological processes, including neurotransmission, olfaction, proliferation and differentiation of neural precursor cells, myelin maintenance, copper and zinc ion transport, and calcium homeostasis, as well as neuroprotective activities against several toxic insults, such as oxidative and excitotoxic damage [2–4]. How can PrP^C^ serve so many different functions? Perhaps the answer lies in its ability to interact with a number of membrane proteins, potentially influencing their cellular localization and activity [4]. These include glutamate receptors of the N-methyl-D-aspartate (NMDA) subclass [5] and voltage-gated calcium channels (VGCCs) [6]. Interaction with these channels may account for some of the functional activities of PrP^C^, but may also activate toxic responses when PrP^C^ misfolds (see below).

## PrP^C^ Mediates PrP^Sc^ Neurotoxicity

The fact that inactivation of the PrP^C^ gene in mice or cows does not cause neurodegeneration indicates that prion pathogenesis is not due to loss of PrP^C^ function, but to a gain of toxicity upon its conversion to PrP^Sc^. Interestingly, extracellular PrP^Sc^ kills only neurons that express PrP^C^. This was first shown by a neurografting experiment in which neural tissue from PrP^C^-expressing mice was transplanted into the brains of PrP knockout mice, which do not replicate prions since they lack the PrP^C^ substrate for PrP^Sc^ production [7]. After intracerebral prion infection, the transplanted mice developed neuropathology in the PrP^Sc^-replicating graft but not in the surrounding PrP knockout tissue, even though this tissue accumulated substantial amounts of graft-derived PrP^Sc^ [8]. Consistently with this, switching off neuronal PrP^C^ expression in mice with established prion infection rescued clinical disease and prevented neuronal loss, despite continuous production of PrP^Sc^ by surrounding astrocytes [9]. Moreover, prion-infected mice expressing a form of PrP^C^ that lacks the GPI anchor and is secreted into the extracellular space did not develop the typical prion pathology despite large amounts of extracellular PrP^Sc^ [10,11]. Thus, PrP^Sc^ is not directly toxic to neurons; it is the endogenous PrP^C^ conversion that causes neuronal dysfunction and death.

Conformational conversion of PrP^C^ starts on the neuronal surface, where PrP^C^ interacts with exogenous PrP^Sc^, and proceeds within endocytic compartments. Thus, neurotoxicity may be triggered by PrP^C^ misfolding at the cell surface or inside the cell.

## Toxicity Induced by PrP^C^ Misfolding at the Neuronal Surface

Two kinds of evidence suggest that alterations in the structure of cell surface PrP^C^ can lead to neuronal death. PrP^C^ molecules with certain internal deletions, including Δ94–134 and Δ105–125, induce dramatic neurodegeneration when expressed in transgenic mice [12,13]. These mutant molecules are efficiently trafficked to lipid raft regions of the plasma membrane, suggesting that their toxicity stems from abnormal activity at the neuronal surface rather than from mislocalization or intracellular retention.

PrP^C^ attenuates the activity of NMDA receptors (NMDARs), protecting neurons from glutamate-induced excitotoxicity [5]. Supporting the idea that the internal deletions may corrupt this function, PrPΔ105–125 sensitized neurons to glutamate-induced, calcium-mediated cell death [14]. It was also found that PrPΔ105–125 induced non-selective ionic currents that depended on the integrity of the N-terminal 23–31 region [15]. A possible interpretation is that the toxic deletions promote a conformational change of the PrP^C^ N terminus, altering its interaction with NMDARs and enabling the 23–31 segment to interact abnormally with the lipid bilayer, generating pores in the plasma membrane. Thus, a structural change in cell surface PrP^C^ would simultaneously corrupt NMDAR function and plasma membrane permeability, leading to dysregulation of ion homeostasis and neuronal death.

Another set of experiments showed that monoclonal antibodies against specific epitopes in the C-terminal globular domain of PrP^C^ induce rapid neurodegeneration when injected into the mouse brain or applied to cultured cerebellar slices [16]. Neurodegeneration was prevented by deleting the PrP^C^ N terminus or by antibodies against this region. The latter also attenuated the toxicity of PrPΔ94–134 [16], suggesting that the globular domain antibodies and the internal deletions activate a similar pathogenic cascade, involving a structural rearrangement of the N terminus.

**Fig 1:**
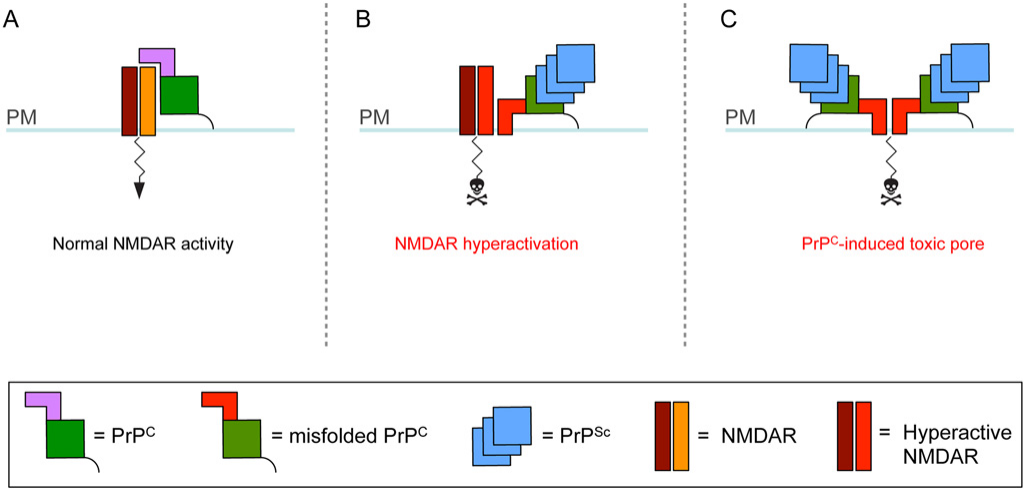
Theoretical model for how cell surface PrP^C^ misfolding could result in neurotoxicity. (A) PrP^**C**^ consists of a flexible N terminus (mauve) and a globular C-terminal domain (green) attached to the plasma membrane (PM) by a GPI anchor (black line). PrP^**C**^ associates with NMDARs, attenuating their activity [5]. (B–C) Interaction with extracellular PrP^**Sc**^ causes the N terminus of PrP^**C**^ to undergo a structural rearrangement. This leads to aberrant interaction of PrP^**C**^ with NMDARs and their hyperactivation (B) and/or abnormal insertion of the PrP^**C**^ N terminus into the lipid bilayer with generation of a toxic pore (C). In addition to NMDARs, PrP^**C**^ misfolding at the cell surface may corrupt the activity of other PrP^**C**^-interacting ion channels or signaling complexes.

Supporting the idea that PrP^Sc^ docking onto cell surface PrP^C^ may elicit a similar structural change and downstream toxic effects (Fig 1), it was found that when PrP^Sc^ was exogenously presented to cultured neurons, the resulting neurotoxicity was blocked by NMDAR antagonists or by deletion of the N-terminal domain of neuronal PrP^C^ [17,18].

## Neurotoxicity Induced by Intracellular PrP^C^ Misfolding

A study in prion-infected mice gave information about a neurotoxic mechanism potentially triggered by intracellular accumulation of misfolded PrP^C^. Prions inoculated into the mouse hippocampus activates the translational repression pathway of the unfolded protein response (UPR). The UPR is a signal transduction cascade set in motion when misfolded proteins accumulate in the ER. A crucial step is auto-phosphorylation of the ER-associated kinase PERK, which phosphorylates the α subunit of the eukaryotic translation initiation factor 2 (eIF2α). This inhibits protein translation, reducing the overload of misfolded proteins. In the case of protracted UPR, however, sustained translational attenuation can have detrimental effects. In prion-infected mice, prolonged activation of the PERK/eIF2α pathway caused drops in the levels of pre- and post-synaptic proteins in the hippocampus, deficits in hippocampal synaptic transmission, and behavioral decline [19].

But what activates the UPR? PrP^Sc^ is unlikely to be the instigating factor, since it accumulates in the extracellular space or in endocytic compartments, rather than in the ER. The level of PrP^C^ mRNA rises during prion infection, and the PrP^C^ mRNA molecules escape eIF2α-P-induced translational inhibition [19]. Thus, ER overload with misfolded PrP^C^ due to increased biosynthesis may be the actual cause of UPR activation. Alternatively, the UPR could be triggered by ER accumulation of ^Ctm^PrP, a transmembrane form of PrP^C^ whose biogenesis at the ER membrane increases in prion-infected mice [20].

**Fig 2:**
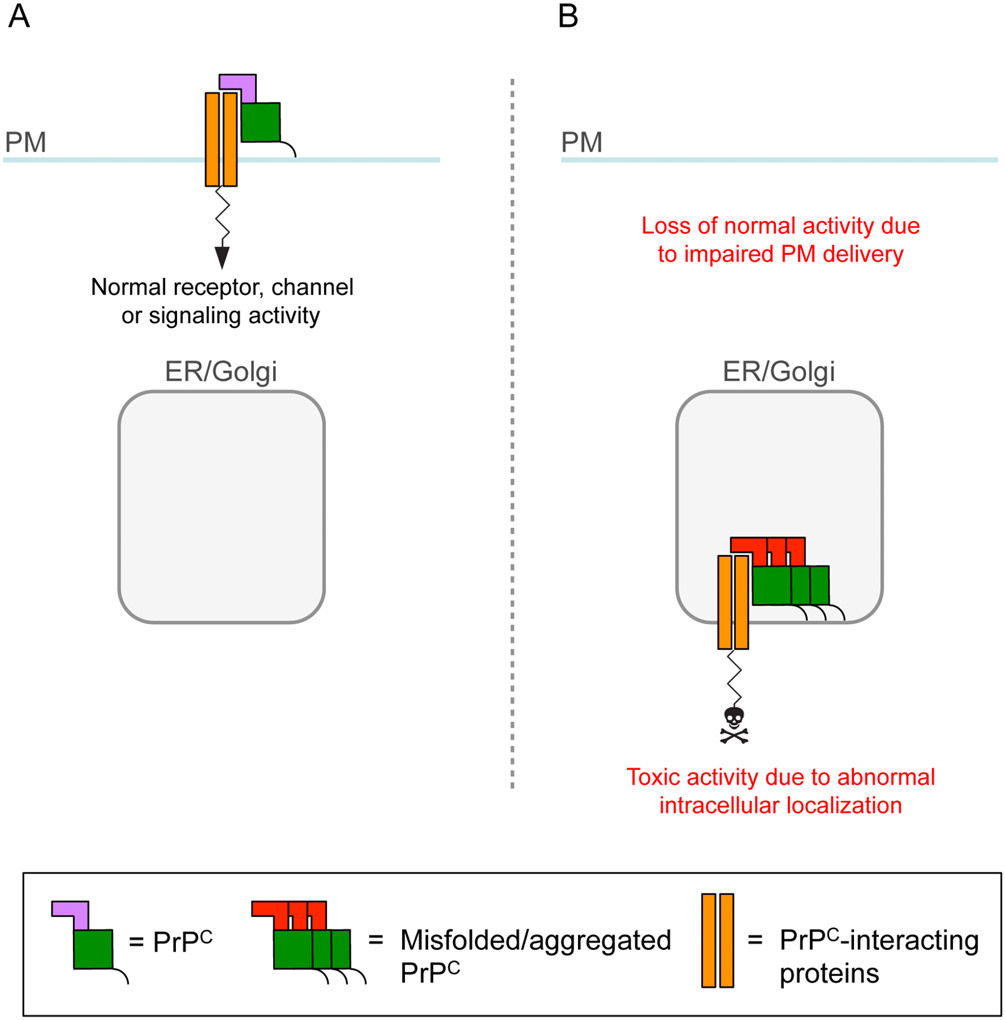
A role for intracellular PrP^C^ retention in neuronal dysfunction. (A) PrP^**C**^ on the plasma membrane (PM) influences the activity of neurotransmitter receptors, ion channels, and signaling complexes with which it interacts. (B) Owing to retention in transport organelles (ER/Golgi), misfolded/aggregated PrP^**C**^ sequesters the interacting protein in intracellular compartments, leading to loss of normal function on the cell membrane [6]. Intracellular retention might also cause the complex to function abnormally and generate a toxic signal.

Prion infections are extremely rare in humans, in whom approximately 99% of all cases occur sporadically or are inherited because of mutations in the gene encoding PrP^C^. In these illnesses, PrP^C^ misfolds spontaneously without the need for contact with exogenous PrP^Sc^. When expressed in transgenic mice, PrP^C^ molecules with certain genetic prion disease-associated mutations cause neurological syndromes that recapitulate key features of the corresponding human disorders [21–23]. These mutant PrPs misfold spontaneously in the ER lumen and are partly retained in the secretory pathway; surprisingly, however, they do not trigger the UPR [24,25]. How do they cause neurological disease? PrP^C^ interacts physically with the α_2_δ-1 subunit of VGCCs [6]. This is a GPI-anchored protein which promotes the anterograde trafficking and correct synaptic localization and function of the channel complex. Owing to ER retention of mutant PrP, α_2_δ-1 accumulates intracellularly, impairing delivery of VGCCs to synapses. This leads to inefficient depolarization-induced calcium influx, abnormal cerebellar neurotransmission, and motor disease [6]. Since PrP^C^ interacts with a number of other proteins that transit the secretory pathway, such as glutamate receptors and signaling complexes, its intracellular retention may have broader effects on neuronal function (Fig 2) [25].

Thus, in both acquired and genetic prion diseases, intracellular PrP^C^ misfolding would ultimately alter synaptic proteostasis, either through an indirect, UPR-mediated mechanism, or by directly interfering with secretory trafficking of PrP^C^-interacting cargoes.

## A Complex Interplay of Extracellular and Intracellular Toxicities

The experimental studies outlined above indicate different neurotoxic mechanisms that may be activated by misfolded PrP^C^ in distinct cellular compartments, including corruption of PrP^C^ interactions on the cell surface, disruption of plasma membrane permeability, impairment of secretory protein transport, and dysregulation of generic proteostatic pathways, such as the UPR.

These mechanisms are likely to co-exist, but may contribute differently to pathogenesis in different prion diseases. UPR-induced transcriptional attenuation may account for the synaptic dysfunction and degeneration that precedes neuronal death in the early stages of prion infection. As the disease progresses and PrP^Sc^ accumulates in the extracellular space, additional mechanisms may be engaged. PrP^Sc^-induced misfolding of cell surface PrP^C^ may be a key mediator of cell death [10] and cause rapid neuron demise by corrupting ion channel or signaling activities, and/or by generating toxic pores (Fig 1). In sporadic and genetic prion diseases, in which PrP^Sc^ formation is not obligatory for pathogenesis [26–28], spontaneous accumulation of misfolded PrP^C^ molecules in transport organelles may be more important. Misfolded/aggregated PrP^C^ may sequester ion channels or signaling complexes in intracellular compartments, leading to loss of their normal functions on the cell membrane and/or gain of toxic intracellular activities (Fig 2) [6].

This neurotoxic modality may contribute to the clinical variability of prion diseases. Different misfolded PrP^C^ variants may be produced in different prion disorders, which may have different effects on neuronal function—hence, on the clinical presentation of disease—depending on their propensity to accumulate in intracellular organelles and interfere with the transport of the molecules with which they interact [25].

In view of their complex pathogenesis, what would be the best therapeutic option for prion diseases? Several compounds inhibit PrP^Sc^ propagation in cultured cells, but show little or no efficacy in vivo, and no therapeutically useful drug is currently available. The “mutability” of prions [29] means that molecules that target PrP^Sc^ can lead to the selection of drug-resistant variants that propagate more efficiently in the presence of the drug [30]. Given the emerging role of PrP^C^ misfolding in neurotoxicity, drugs that stabilize its native conformation or down-regulate its expression may prove more effective, and applicable to the sporadic, genetic, and acquired forms.
